# The Efficacy and Safety of Minimally Invasive Glaucoma Surgery for Primary Open-Angle Glaucoma: A Systematic Review

**DOI:** 10.3390/healthcare14030319

**Published:** 2026-01-27

**Authors:** Jill Gottehrer, Pinakin Gunvant Davey

**Affiliations:** 1 Department of Veterans Affairs Finger Lakes Healthcare System, Rochester, NY 41623, USA; jill_gottehrer@alumni.neco.edu; 2 College of Optometry, Western University of Health Sciences, Pomona, CA 91766, USA

**Keywords:** blindness, drainage, efficacy, glaucoma, minimally invasive glaucoma surgery, ocular surgery, open-angle, safety, ophthalmology, trabeculectomy, treatment outcome

## Abstract

**Highlights:**

**What are the main findings?**

- Minimally invasive glaucoma surgeries (MIGSs) and implants serve as a safe and effective option to lower intraocular pressure.
- The lowering of intraocular pressure with MIGS is comparable to other invasive procedures and surgeries that are proven in the management of glaucoma.

**What is the implication of the main finding?**

- The healthcare system may benefit from the widespread use of MIGS, as the training needed is minimal and the recovery time post-surgery is as short as that for cataract extraction.
- It is obvious that MIGSs are going to remain important in the foreseeable future, and healthcare systems will need to invest resources in studying their long-term safety and efficacy.

**Abstract:**

Healthcare systems worldwide are burdened significantly due to glaucoma, which is a leading cause of irreversible blindness. A total of 76 million are currently affected, and it is estimated that by the year 2040, 112 million will be affected by glaucoma. Minimally invasive glaucoma surgery (MIGS) is a recent innovation that plays a key role in glaucoma management. We aimed to conduct a systematic review of the safety and efficacy of MIGS devices and procedures and their use in primary open-angle glaucoma (POAG) to lower intraocular pressure (IOP). **Methods:** A comprehensive electronic search of the PubMed database was conducted, and randomized controlled trials (RCTs) comparing MIGS devices and techniques with cataract surgery or other glaucoma procedures that had been published by 1 May 2025 were included. **Results:** Thirty RCTs were included in the systematic review. Studies show that MIGSs are as safe and effective as other procedures, including phacoemulsification and trabeculectomy, at lowering IOP. **Conclusions:** Short-term trials indicate that MIGSs are a safe and effective treatment option for primary open-angle glaucoma. MIGS procedures lead to favorable outcomes, including decreases in mean IOP and medication use, compared with other glaucoma procedures or standalone phacoemulsification. Independent long-term follow-up studies are needed further to elucidate the efficacy and long-term safety of MIGS.

## 1. Introduction

Glaucoma is an optic neuropathy that leads to irreversible and progressive vision loss that affects more than 70 million individuals worldwide [1,2]. It is estimated that 50 million people have primary open-angle glaucoma (POAG), with an estimated 4.14 million people with moderate-to-severe visual impairment and 3.6 million suffering from blindness globally [3,4]. Elevated intraocular pressure (IOP) is the most significant and the only proven modifiable risk factor. The primary treatment strategy for POAG aims to minimize glaucomatous damage by lowering IOP [5]. Although first-line glaucoma management includes ocular hypotensive medications, a myriad of factors influence adherence to and persistence with the treatment regimen. Such factors include dosing with multiple medications, difficulty with drop instillation, side effects, and cost [6,7].

Surgical intervention is often considered when topical therapy or other treatment, such as laser, is inadequate at lowering the IOP, and there is a substantial risk of disease progression. Surgical approaches for glaucoma include trabeculectomy, ab externo filtration, incisional surgery, and tube shunt surgery. However, conventional glaucoma filtering surgeries like trabeculectomy, while effective at lowering the IOP, have the potential for intraoperative and postoperative complications compared to tube shunts (41% vs. 29, respectively) [8]. These include serious complications such as hypotony (abnormally low IOP), infection, and suprachoroidal hemorrhage, which may require additional surgeries [9]. Additionally, more invasive surgeries lead to greater accelerated cataract formation when compared to IOP-lowering medications. Furthermore, the fellowship in glaucoma specialty ophthalmology training is needed to perform these complicated procedures.

The development of minimally invasive glaucoma surgery (MIGS) represents a significant advancement in glaucoma management, leading to safer, less invasive interventions without the side effects and the need for daily applications of topical therapy. The various MIGS devices are suitable in the management of a wide variety and stages of glaucoma, from early to more refractory [10]. The term MIGS was first introduced by Professor Ike Ahmed in 2009. The goals of such procedures and devices were that they had five key features: (1) an ab interno approach, (2) minimal disruption of ocular anatomy, (3) highly efficacious approach providing a highly safe and rapid recovery [11]. The advantages of an ab interno approach, which is typically performed through clear corneal incision, is the decrease in complications such as infections, scarring, and hypotony. This has a higher safety profile and may be interesting to general ophthalmologists for combined procedures during cataract surgery. MIGSs can be further categorized based on their mechanism of action and surgical approach: enhancing trabecular outflow (Trabectome, iStent, iStent inject, Hydrus Microstent, Kahook Dual Blade Goniotomy, and Gonioscopy-Assisted Transluminal Trabeculotomy), procedures aimed at Schlemm’s canal (TRAB360, OMNI, and ab interno canaloplasty), augmentation of suprachoroidal flow (CyPass Microstent and iStent Supra), cycloablative procedures (endocyclophotocoagulation and micropulse cyclophotocoagulation), and conjunctival bleb-forming procedures (XEN Gel Stent, EX-PRESS Glaucoma Filtration Device, and PreserFlo MicroShunt).

Cataracts are another leading cause of blindness worldwide. In 2020, an estimated 42.3 million people were blind, and 295 million had moderate–severe visual impairment due to cataracts [12]. Both cataracts and glaucoma are age-related diseases; they often coexist in the same eye. Against this backdrop, it is important to understand the role that primary care providers and other healthcare providers play in encouraging routine ophthalmological evaluations and in appropriately explaining the risks and benefits of various management strategies to help prevent irreversible blindness and improve quality of life.

This systematic review primarily aims to provide an up-to-date summary of the current evidence of MIGSs, elaborating on their different underlying principles, mechanisms, safety, and efficacy in POAG patients over the last twenty years. We will discuss the various MIGS devices and techniques, including trabecular micro-bypass stents, suprachoroidal shunts, and subconjunctival filtration devices. Additionally, we will discuss the advantages and limitations of each MIGS device. Lastly, we will discuss the future of MIGS devices/procedures.

### 1.1. The Need for MIGS

Prior to the emergence of MIGS, glaucoma surgery was dominated by filtering surgeries, characterized by their invasiveness and risk of complications [13]. Trabeculectomy was first introduced in the 1960s and was considered the gold standard of glaucoma surgery for decades. While effective at lowering the IOP, the success of trabeculectomy is dependent on surgical skill/expertise and is associated with severe postoperative complications such as hypotony due to multiple variables [14].

Procedures such as cyclocryotherapy and cyclophotocoagulation were developed to lower IOP by the destruction of ciliary processes in the ciliary body that are responsible for aqueous humor production. Given that these procedures were performed from the outside without visualization of the structure, they often led to over-treatment and were prone to various complications. The complications included phthisis bulbi (atrophy of the globe), chronic inflammation, and loss of vision [14]. Thus, rightfully so, these procedures were reserved for advanced glaucoma or eyes that were blind but painful due to elevated IOP.

Glaucoma drainage devices such as Ahmed, Baerveldt, and Molteno were alternatives in cases when trabeculectomies were less likely to be successful, such as neovascular or uveitic glaucoma. While effective at lowering the IOP, traditional glaucoma tube surgeries were often associated with a plethora of complications that included tube exposure, erosion, blockage, migration, and hypotony [14]. These procedures required extensive postoperative care, including frequent follow-up visits, creating a burden on patients/caregivers. This situation created the need for less invasive and safer surgical options; hence, it paved the way for the development of MIGS.

Early MIGS devices began with the concept of enhancing aqueous outflow by bypassing the trabecular meshwork and Schlemm’s canal. Approved by the FDA in 2004, Trabectome (Microsurgical Technology, Redmond, WA, USA) was one of the first MIGS devices that was able to achieve this goal [15]. An additional early innovation in the MIGS field was the iStent (Glaukos, Laguna Hills, CA, USA), a device to be implanted in Schlemm’s canal to facilitate aqueous outflow. It received FDA approval in 2012 to be used in conjunction with phacoemulsification (cataract surgery). These devices set the stage for new options for glaucoma patients, especially those who were at a high risk for complications from traditional filtering surgeries.

### 1.2. Impact on Glaucoma Management Practices

MIGS has created a paradigm shift from medical management of glaucoma being the first choice to “interventional glaucoma” surgeries being desired that are less invasive and safer. They also decreased the burden related to the need for multiple medications to achieve target IOP, potentially shifting the trajectory of glaucoma progression. Additionally, the relatively easier procedural technique, less invasive approach, higher safety profile, and ability to be performed at the same time as cataract surgery (phacoemulsification) may lead to higher patient acceptance of surgical intervention compared to traditional filtering surgeries [16].

## 2. Materials and Methods

A systematic review of the literature was conducted using the PubMed database to identify relevant MIGS studies. The search strategy involved the utilization of the following keywords and terms: *MIGS, microinvasive glaucoma surgery, and minimally invasive glaucoma surgery.* The search period was set from 1 January 2005 to 1 May 2025 to capture the most up-to-date evidence. Conference abstracts usually lack the detailed analysis and information and were not included and publications in languages other than English were excluded. This review focused on POAG, which is the most common type of glaucoma. Glaucoma subtypes other than primary open-angle glaucoma were also excluded as the mechanisms of the disease process vary and MIGS are not designed for these subtypes. This systematic review does not involve human subjects and is exempt from review by the Institutional Review Board. This systematic review is registered on PROSPERO (registration number: CRD420251178182).

The Cochrane Collaboration’s tool [17] for assessing risk of bias was utilized in the study to assess overall completeness of data reporting (see Appendix A). The studies included in this systematic review had very low risk of bias due to random sequence generation, incomplete information of data, and selective reporting. However, not all studies had a clear explanation or sufficient data on allocation concealment. Furthermore, many trials were pivotal trials submitted to the Food and Drug Administration (FDA) for device clearance and approval and therefore funded by the manufacturer. Some investigators involved in the study were sometimes consultants and employees of the company, and in those cases, the studies were categorized in other risk of bias category.

**Figure 1:**
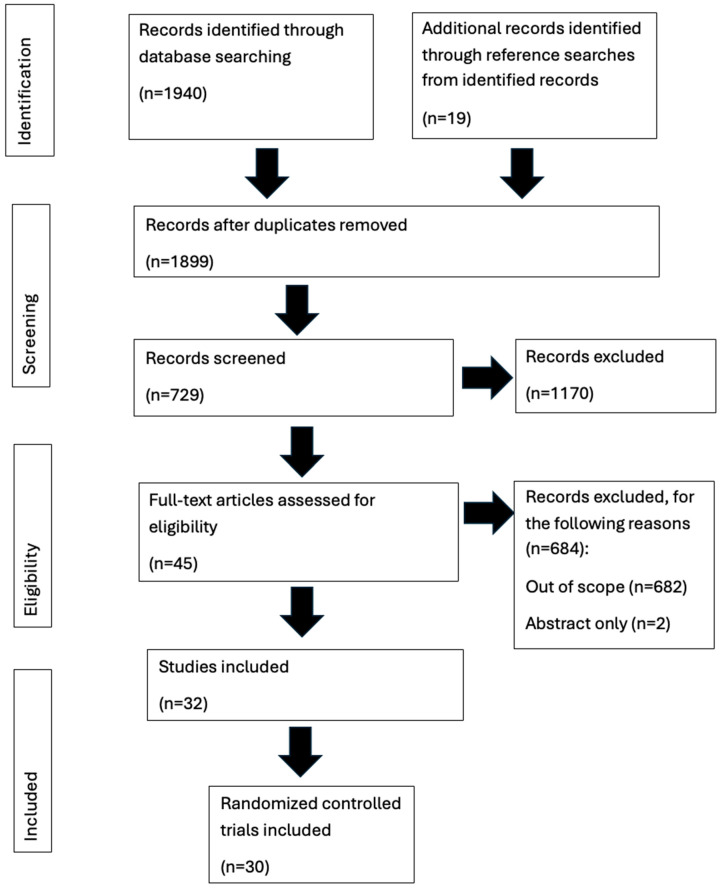
PRISMA flow chart, outlining the systematic review of MIGS devices and procedures in primary open-angle glaucoma.

Figure 1 provides PRISMA flow chart, outlining the systematic review showing the articles screened and finally included in the systematic review.

## 3. Results

### 3.1. Bypassing Trabecular Meshwork by Implantable Devices

The trabecular meshwork is considered one of the primary sources of resistance to aqueous humor outflow. The trabecular outflow accounts for 70% to 90% of total aqueous outflow [18]. In glaucoma, the trabecular meshwork becomes blocked due to factors such as increased resistance and cell necrosis. The goal of trabecular bypass is to improve IOP control by creating a new drainage pathway [19]. Current MIGS trabecular micro-bypass devices include the iStent and Hydrus.

The iStent (Glaukos, Laguna Hills, CA, USA) was the first-generation single nonferromagnetic titanium stent, featuring a 40-degree angled inlet connected to a heparin-coated intracanalicular half-cylinder to minimize fibrosis and enhance aqueous outflow [20]. This may be used as a standalone procedure or combined with cataract surgery with phacoemulsification in those with mild–moderate POAG. If the iStent is implanted as a standalone procedure, a 1.5–2 mm clear corneal incision is made temporally. If the iStent is implanted during phacoemulsification, then the same corneal incision is used. The iStent penetrates the trabecular meshwork and resides in Schlemm’s canal. The most common adverse effects following iStent implantation include an IOP spike, stent blockage or obstruction, stent malposition, hyphema (blood in the anterior chamber), and blood reflux [21]. Table 1 provides the details of iStent randomized controlled trials.

The second-generation stent, iStent inject (Glaukos, Laguna Hills, CA, USA), featured two button-shaped titanium stents that reside in Schlemm’s canal, a thorax that resides in the trabecular meshwork, and a rear flange that resides in the anterior chamber to enhance IOP reduction [22]. This device may also be used as a standalone procedure or in combination with phacoemulsification in patients with mild-to-moderate POAG. The iStent inject is implanted in the eye similarly to the first-generation stent. A key difference between the first-generation iStent and the second-generation iStent inject is that two stents can be implanted with the same inserter in the iStent inject. Transient IOP elevation and hyphema have been reported as postoperative complications [23]. Fea et al. [24] demonstrated in a randomized controlled trial the cost-effectiveness of the iStent inject compared to phacoemulsification alone. They further demonstrated a moderate quality of life gain in those who received the iStent inject and phacoemulsification [24]. Table 2 provides the details of iStent inject randomized controlled trials.

**Table 1:** Characteristics and methodology of iStent randomized controlled trials.

Study	Number of	Age of	Length of	Baseline IOP	IOP-Lowering Effect	Secondary	Efficacy/
(Name)	Participants	Participants (Years)	Study (Months)	(mmHg)	(mmHg)/Outcome	Outcome	Rate of Success
Ahmed, 2020 [25]	75	66.9 ± 10.0	12	19.0 ± 3.9	17.3 ± 3.3	46.6% (medication	30.1% (IOP ≤ 18 mmHg
(COMPARE)	(Hydrus)	(Hydrus)		(Hydrus)	(Hydrus)	reduction; Hydrus)	without treatment; Hydrus)
	77	66.5 ± 9.5		19.1 ± 3.6	19.2 ± 2.4	24% (medication	9.3% (IOP ≤ 18 mmHg
	(iStent)	(iStent)		(iStent)	(iStent)	(reduction; iStent)	without treatment; iStent)
Ahmed, 2024 [26]	69 ± 7.8 (iStent	178 (iStent	60	not reported	not reported	2099 ± 430	not reported
	inject treatment)	inject treatment)				(ECL cells/mm^2^)	
	69.3 ± 7.0	49		not reported	not reported	2103 ± 419	not reported
	(iStent inject control)	(iStent inject control)				(ECL cells/mm^2^)	
	71.1 ± 7.9	369		24.4 ± 2.8	not reported	1967 ± 522	not reported
	(Hydrus treatment)	(Hydrus treatment)		(Hydrus treatment)		(ECL cells/mm^2^)	
	71.2 ± 7.6	187		24.5 ± 3.0	not reported	2117 ± 442	not reported
	(Hydrus control)	(Hydrus control)		(Hydrus control)		(ECL cells/mm^2^)	
	69.4 ± 7.9	215		24.5 ± 2.9	not reported	1931 ± 517	not reported
	(CyPass treatment)	(CyPass treatment)		(CyPass treatment)		(ECL cells/mm^2^)	
	70.8 ± 7.5	67		24.8 ± 3.1	not reported	2189 ± 375	not reported
	(CyPass control)	(CyPass control)		(CyPass control)		(ECL cells/mm^2^)	
Katz, 2015 [27]	38	68.1 ± 9.1	18	19.8 ± 1.3	15.9 ± 0.9)	not reported	89.2% (IOP ≤ 18 mmHg
	(one stent)	(one stent)		(one stent)	(one stent)		unmedicated; one stent)
	41	67.8 ± 9.3		20.1 ± 1.6	14.1 ± 1.0	not reported	90.2% (IOP ≤ 18 mmHg
	(two stents)	(two stents)		(two stents)	(two stents)		unmedicated; two stents)
	40	60.9 ± 8.1		20.4 ± 1.8	12.2 ± 1.1	not reported	92.1% (IOP ≤ 18 mmHg
	(three stents)	(three stents)		(three stents)	(three stents)		unmedicated; three stents)
Samuelson, 2011 [28]	117	73	12	18.4 ± 3.2	1.5 ± 3.0	not reported	72% (≥20% IOP reduction;
	(iStent + CE)	(iStent + CE)		(iStent + CE)	(iStent + CE)		iStent + CE)
	123	73		18.4 ± 3.2	1.0 ± 3.3	not reported	50% (≥20% IOP reduction;
	(CE)	(CE)		(CE)	(CE)		CE)
Craven, 2012 [29]	116	not specified	24	25.4 ± 3.6	17.0 ± 2.8	not reported	53% (≥20% IOP reduction;
	(iStent + CE)			(iStent + CE)	(iStent + CE)		iStent + CE)
	123	not specified		25.2 ± 3.6	17.0 ± 3.1	not reported	44% (≥20% IOP reduction;
	(CE)			(CE)	(CE)		CE)

Legend: CE: cataract extraction, ECL: endothelial cell loss.

**Table 2:** Characteristics and methodology of iStent inject randomized controlled trials.

Study	Number of	Age of	Length of	Baseline IOP	IOP-Lowering Effect	Secondary	Efficacy/Rate of Success
(Name)	Participants	Participants (Years)	Study (Months)	(mmHg)	(mmHg)/Outcome	Outcome	>20% IOP Reduction
Fan, 2024 [30]	56	73.9 ± 7.4	24	17.7 mmHg ± 4.0	0.7 ± 0.9 glaucoma medications	Improvement in VF QofL in both groups	not reported
	(iStent inject + CE)	(iStent inject + CE)		(iStent inject + CE)	(iStent inject + CE)	compared to baseline	
	48	72.6 ± 7.7		17.1 mmHg ± 3.1	1.5 ± 1.9 glaucoma medications	Improvement in VF QofL in both groups	not reported
	(CE)	(CE)		(CE)	(CE)	compared to baseline	
Lima, 2023 [31]	35	66.2 ± 9.3	12	22.1 ± 3.6	↓24.2%	not reported	not reported
	(ECP + CE)	(ECP + CE)		(ECP + CE)	(ECP + CE)		
	36	68.7 ± 6.9		22.0 ± 2.5	↓43.6%	not reported	not reported
	(ECP + CE + iStent inject)	(ECP + CE + iStent inject)		(ECP + CE + iStent inject)	(ECP + CE + iStent inject)		

Legend: CE: cataract extraction, ECP: endoscopic photocoagulation, VF QofL: Visual function quality of life.

**Figure 2:**
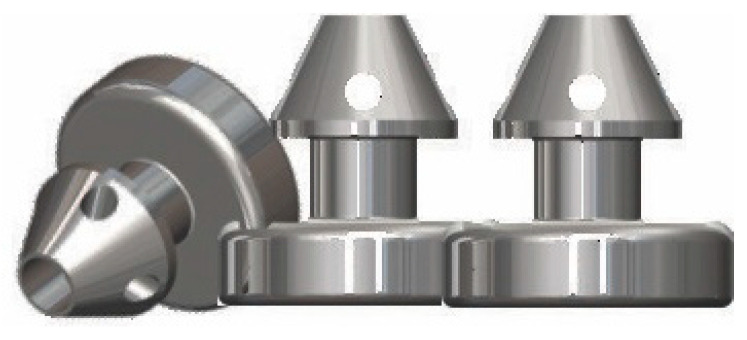
iStent inject W—provided courtesy of Glaukos.

The third-generation stent, iStent inject W (Glaukos, Laguna Hills, CA, USA), features a wider flange at its base to optimize stent visualization and placement. This device (Figure 2) features two preloaded stents that can be implanted through a single entry point. This may be a standalone procedure or combined with phacoemulsification in those with mild-to-moderate POAG. During this procedure, the surgeon implants the stent through a clear corneal incision, which is made in the temporal cornea (same location as cataract extraction) to place the stents in the nasal aspect of Schlemm’s canal. Transient IOP elevation and hyphema have been reported as postoperative complications [23].

The VENICE study was a randomized controlled trial comparing the safety and efficacy of iStent inject W to STREAMLINE in mild-to-moderate primary open-angle glaucoma patients undergoing phacoemulsification. A total of 72 eyes were randomized, 35 underwent STREAMLINE canaloplasty, and 37 were implanted with the iStent inject W. Baseline IOP in the STREAMLINE group was 24.86 ± 3.05 mmHg compared to 25.16 ± 3.41 in the iStent W group, with a mean IOP at 6 months of 16.52 ± 3.63 in the STREAMLINE group compared to 16.08 ± 3.19. This difference in postoperative IOP between the two procedures was not statistically significant. At 6 months, more eyes were on no glaucoma medications in the STREAMLINE group (81.8%) compared to the iStent W group (78.4%). The authors also concluded that the adverse effects were mild and self-limiting [32].

**Figure 3:**
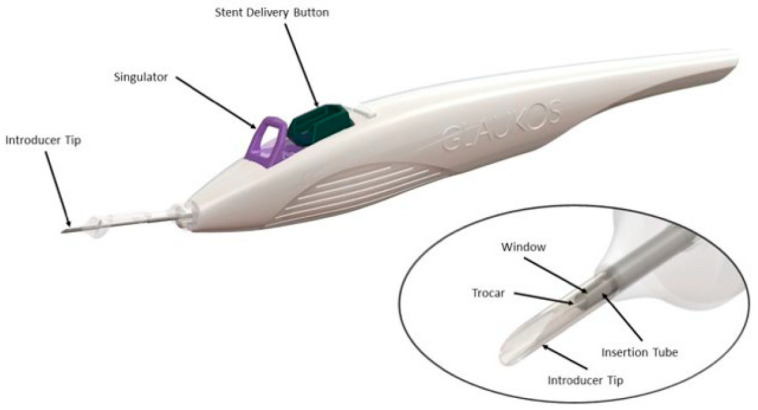
iStent Infinite, picture provided courtesy of Glaukos.

Comparatively, iStent Infinite (Glaukos, Laguna Hills, CA, USA) is intended to be a standalone procedure for those who previously failed other medical and surgical treatments. The name “infinite” was derived due to there being no limit to the number of times a stent can be deployed compared to other iStent models. This device (Figure 3) consists of three microscale wide-flange stents on a single preloaded injector to be injected over three separate areas of the trabecular meshwork, creating a patent bypass through the trabecular meshwork into Schlemm’s canal to increase physiological outflow and reduce IOP. A clear corneal incision is made, and the stents are injected into the trabecular meshwork. As there are three stents, they should be placed approximately two–three clock hours apart. The most common postoperative complications include an IOP increase ≥ 10 mmHg, loss of best-corrected visual acuity ≥ 2 lines, ocular surface disease, perioperative inflammation, and visual field loss ≥ 2.5 decibels [33]. There are no published randomized controlled trials for this device.

The Hydrus Microstent (Ivantis Inc, Irvine, CA, USA) consists of nonferromagnetic nickel–titanium alloy and incorporates a curved, flexible open design to dilate approximately 8 mm of Schlemm’s canal to improve aqueous outflow and bypass the trabecular meshwork [34]. An injector that is preloaded with the device is utilized to insert the device into the Schlemm’s canal. Although the injector aids in appropriate placement of the device, it has to be remembered that incorrect placement, mispositioning, and unexpected postoperative shifts may still occur. The Hydrus Microstent may be implanted as a standalone procedure or in combination with phacoemulsification in patients with mild-to-moderate primary open-angle glaucoma. This device is inserted via a temporal clear corneal incision followed by implantation of the stent into the nasal Schlemm’s canal spanning approximately 90 degrees. Postoperative complications may include device obstruction, obstruction of the microstent inlet by peripheral anterior synechiae (adhesions between the iris and the trabecular meshwork), transient hyphema, and an IOP spike [35,36]. Table 3 summarizes the Hydrus Microstent studies.

Both the iStent and Hydrus have shown successful lowering of IOP when performed with phacoemulsification when compared to a traditional standalone phacoemulsification procedure. The Compare Study [25] was a prospective, randomized, single-masked trial that compared the efficacy of the iStent to Hydrus. A total of 152 eyes of 152 patients aged 45 to 84 with open-angle glaucoma were enrolled. Eyes were randomized to standalone MIGS consisting of either one Hydrus Microstent or two iStent devices. Patients were evaluated at 1 day, 1 week, and 3, 6, and 12 months postoperatively. The authors found that by 1 year, the Hydrus stent implantation group required less medication usage compared to the iStent group (46.6% vs. 24.0%). At 12 months, the Hydrus group eliminated 1.6 medications compared to 1.0 in the iStent group. The mean IOP in the Hydrus group was 17.3 ± 3.7 mmHg with 39.7% of patients achieving ≥20% IOP reduction. Comparatively, the mean IOP in the iStent group was 19.2 ± 2.4 mmHg with 13.3% of patients achieving ≥20% IOP reduction [25].

### 3.2. Bypassing the Trabecular Meshwork by Tissue Excision: Enhancing Aqueous Outflow Through Schlemm’s Canal

Schlemm’s canal: Schlemm’s canal is a key source of outflow resistance leading to increased IOP. A smaller fraction of aqueous humor drains through the non-trabecular outflow pathway. Here, the aqueous humor flows through the ciliary muscle into the suprachoroidal space [37]. As the trabecular meshwork is considered the primary site of resistance to aqueous humor outflow, bypassing this structure and directing aqueous flow directly from the anterior chamber into Schlemm’s canal is an approach that can enhance aqueous outflow.

Kahook Dual Blade Goniotomy (New World Medical, Rancho Cucamonga, CA, USA) is a single-use instrument designed to stretch and remove a strip of trabecular meshwork. Through a temporal clear corneal incision, the instrument pierces the trabecular meshwork. Next, two parallel blades excise a strip of the trabecular meshwork, allowing for unobstructed flow of the aqueous humor into Schlemm’s canal and minimal collateral damage to the surrounding tissue. This contributes to faster recovery times and fewer complications [38]. This strip is then removed from the eye by either forceps or the instrument itself. This procedure may be performed as a standalone procedure or with phacoemulsification in those with moderate-to-severe primary open-angle glaucoma. Common postoperative complications include transient hyphema and an IOP spike [39].

Arimura et al. [40] found in a randomized controlled trial using anterior segment optical coherence tomography that the Kahook Dual Blade Goniotomy group had a larger incisional cross-sectional area in the trabecular meshwork compared to the microhook group throughout the postoperative follow-up visits. Compared to Kahook Dual Blade Goniotomy, microhook ab interno trabeculectomy incises the trabecular meshwork using small hooks that are inserted through corneal side ports. It was theorized that this contributes to reduced trabecular meshwork repair. However, there was no statistically significant difference in postoperative IOP or the number of glaucoma medications between the two groups providing favorable data in favor of the microhook ab interno device [40]. 

Ventura et al. [40] compared Kahook Dual Blade Goniotomy with cataract extraction in a single-center longitudinal, randomized controlled trial. A total of 43 eyes with open-angle glaucoma or ocular hypertension were randomized, 22 to Kahook Dual Blade Goniotomy and cataract extraction and 21 to cataract extraction alone. Baseline IOP in the Kahook Dual Blade Goniotomy and cataract extraction group was 17.3 ± 3.5 mmHg compared to 17.3 ± 3.5 mmHg in the cataract extraction alone group. At 12 months, the mean IOP in the Kahook Dual Blade Goniotomy and cataract extraction group was 16.0 ± 2.2 mmHg compared to 15.0 ± 3.2 mmHg in the cataract extraction alone group. The authors also concluded that both groups had similar best-corrected visual acuity, endothelial cell counts, and standard automated perimetry results [41].

Trabectome (Microsurgical Technology, Redmond, WA, USA) is a microelectrocautery device that applies high-frequency electrical energy. This electrical energy ablates and extracts a strip of trabecular meshwork along with the inner wall of Schlemm’s canal, allowing aqueous humor outflow directly into the canal [15]. Although this procedure causes decreased damage to the tissues and decreased inflammation and postoperative scarring compared to other procedures, it should be noted that microcautery procedures using high-frequency ablation may lead to intraoperative blood reflux. Spontaneous blood reflux does not necessarily affect postoperative outcome, but it may increase intraoperative visualization, possibly prolong surgical duration, and increase surgical difficulty. Other adverse effects of Trabectome may include transient hyphema and elevated IOP on the first post-op day. Trabectome may be performed as a standalone procedure or in conjunction with phacoemulsification. It is most beneficial in those with moderate-to-severe primary open-angle glaucoma [42]. Ting et al. [43] compared Trabectome with cataract surgery (phaco-AIT) to trabeculectomy with mitomycin C and cataract extraction (phaco-trabeculectomy) in a prospective randomized controlled trial with a single surgeon and surgical center. A total of 19 eyes with open-angle glaucoma were randomized, 10 to phaco-AIT and 9 to phaco-trab. The baseline IOP was 20.0 ± 5.3 mmHg in the phaco-AIT group compared to 23.1 ± 6.4 mmHg in the phaco-trab group. After 12 months, the mean IOP was 17.5 ± 3.8 mmHg in the phaco-AIT group with a mean medication reduction of 0.44 ± 0.88. Comparatively, the mean IOP in the phaco-trab group was 16.0 ± 6.0 mmHg, with a mean medication reduction of 0.75 ± 0.89. Efficacy was defined as a reduction in IOP greater than 20%. A total of 40% of eyes with phaco-AIT achieved this compared to 87.5% in the phaco-trab group. The authors concluded that both groups achieved similar IOP that was lower at 6 and 12 months and a similar number of glaucoma medications required at 1 year [43].

Gonioscopy-Assisted Transluminal Trabeculotomy (NeoMedix Co., Tustin, CA, USA) (GATT) is performed by an ab interno approach. This procedure involves a 360-degree trabeculectomy using either a microcatheter or a Prolene suture to aid in the circumferential unroofing of Schlemm’s canal [44]. The GATT procedure is performed through a temporal clear corneal incision, followed by the insertion of the microcatheter within Schlemm’s canal under gonioscopic guidance, which is carefully threaded circumferentially around the canal. Once the microcatheter tip is visualized in the opposite side of the anterior chamber angle, the suture is tied to the microcatheter and subsequently drawn through the length of Schlemm’s canal. Next, the suture is gently pulled, essentially unroofing the trabecular meshwork and creating a trabeculotomy of up to 360 degrees. GATT may be performed as a standalone procedure or with phacoemulsification. This procedure is indicated for those with moderate-to-severe POAG. Transient hyphema is the most common postoperative complication [45]. Yin et al. [46] compared the efficacy and safety of ab interno canaloplasty (ABiC) with GATT in a randomized controlled trial. Thirty-eight eyes with POAG were randomized to ABiC and twenty-nine to GATT. Follow-ups were performed at 1, 3, 6, and 12 months postoperatively. The primary outcome measures were IOP and use of glaucoma medication at 12 months postoperatively. The second outcome measure was complete surgical success, defined as an IOP ≤ 21 mmHg without requiring glaucoma medications and not needing additional glaucoma surgery. At 12 months, the mean IOP was 19.0 ± 5.2 mmHg in the ABiC group and 16.0 ± 3.1 mmHg in the GATT group. In the ABiC group, 57.2% of patients were medication-free compared to 75% in the GATT group. Three eyes in the ABiC group needed additional glaucoma surgery compared to one eye in the GATT group. The authors concluded that GATT was advantageous over ABiC in terms of IOP reduction in primary open-angle glaucoma patients [46].

### 3.3. Enhancing Aqueous Outflow Through Schlemm’s Canal

**Figure 4:**
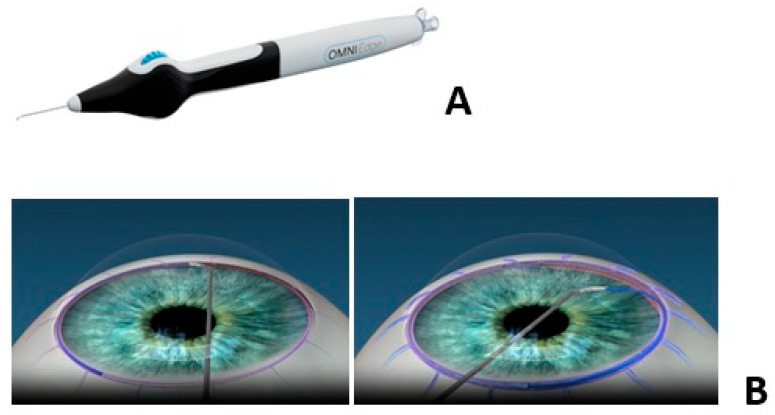
OMNI device images (**A**) and canaloplasty (**B**) and trabeculotomy animation (provided courtesy of Sight Sciences). The blue line in the picture represents the catheter that’s introduced into the Schlemm’s canal for the release of viscoelastic material and performing trabeculectomy.

The OMNI device (Sight Sciences, Menlo Park, CA, USA) is a single-use device designed to enhance aqueous outflow that performs both canaloplasty and trabeculotomy. The OMNI device (Figure 4) dilates Schlemm’s canal and its collector channels. Through a temporal clear corneal incision, the device is forwarded into the anterior chamber and the nasal Schlemm’s canal is accessed. A microcatheter is inserted nasally and advanced, which automatically that moves circumferentially due to the Schlemm’s canal’s anatomy. Upon retraction, a viscoelastic is dispensed at approximately 5.5 microliters per 20 mm advancement. This procedure is performed a second time without retracting the microcatheter that performs the trabeculotomy [47]. OMNI may be performed as a standalone procedure or with phacoemulsification in those with mild-to-moderate primary open-angle glaucoma. Postoperative complications may include hyphema, iridodialysis, cyclodialysis, and partial goniosynechiae [48]. No randomized controlled trials have been found for this device.

TRAB360 (Sight Sciences, Menlo Park, CA, USA) is a single-use device that allows a trabeculotomy to be performed through a temporal clear corneal incision. Next, the trabecular meshwork is incised with the tip of the device, and the microcatheter is advanced from the tip and threaded through 180 degrees of Schlemm’s canal to unroof the trabecular meshwork to achieve 180 degrees of trabeculotomy. Once deployed, the device is retracted, then rotated and reinserted. The procedure is repeated for the remaining 180 degrees of the angle to accomplish a complete 360-degree treatment. This device is recommended for those with mild-to-moderate primary open-angle glaucoma, either as a standalone procedure or in conjunction with phacoemulsification. There are no published randomized controlled trials for this device at this time.

The iTrack microcatheter system (Ellex iScience, Fremont, CA, USA) can be used to perform either ab externo or ab interno canaloplasty. The device uses an illuminated fiber-optic light 250 μm microcatheter to perform the procedure. The illumination allows visualization of Schlemm’s canal during the procedure whilst the catheter is advanced 180 degrees in each direction. The viscoelastic material is released from the tip of the catheter, which is retracted by the surgeon. The purpose of the viscoelastic material is to dilate Schlemm’s canal, which aids restoration of conventional outflow [49]. This device and procedure can be performed during cataract surgery or as a standalone procedure and is recommended for mild-to-moderate glaucoma. Ye et al. [50] compared penetrating canaloplasty to ab externo canaloplasty (CP) in a randomized controlled trial. A total of 52 patients with open-angle glaucoma were randomized: 26 to penetrating canaloplasty and 26 to CP. The baseline IOP in the penetrating canaloplasty group was 30.8 ± 10.7 mmHg compared to 28.6 ± 11.8 mmHg in the CP group. After 24 months, the mean IOP was 14.1 ± 3.3 mmHg in the penetrating canaloplasty group with a mean number of glaucoma medications of 0.2 ± 0.5 compared to 22.1 ± 13.6 mmHg in the CP group with a mean number of medications of 0.7 ± 1.2. Efficacy was defined as an IOP reduction ≥20%; 92% of the penetrating canaloplasty group achieved this compared to 77% of the CP group [50]. The authors concluded that compared to ab externo canaloplasty, penetrating canaloplasty had a greater surgical success rate and IOP reduction. Table 4 summarizes the characteristics and methodology of canaloplasty randomized controlled trials.

The STREAMLINE system (New World Medical, Rancho Cucamonga, CA, USA) enhances aqueous outflow by accessing Schlemm’s canal through a canaloplasty-based approach. This technique uses cannulation and viscodilation of the canal without performing a trabeculotomy. The device is a single-use stainless steel inner cutting cannula encased in a polymer outer sleeve, introduced through a clear corneal incision.

As the surgeon retracts the outer sleeve, the instrument creates a 150 µm goniotomy while simultaneously delivering roughly 7 µL of viscoelastic into Schlemm’s canal. This viscodilation expands both the canal and its collector channels, thereby improving aqueous humor egress and contributing to IOP reduction [27]. STREAMLINE can be incorporated into phacoemulsification or be performed as a standalone procedure in those with mild-to-moderate primary open-angle glaucoma. No significant postoperative outcomes such as IOP spikes or hyphema have been observed [51].

### 3.4. Ab Interno Suprachoroidal/Supraciliary Devices

#### Augmentation of Suprachoroidal Flow

The space between the sclera and the choroid and the sclera and the ciliary body is known as the suprachoroidal and the supraciliary space, respectively. The uveoscleral pathway utilizes this region for outflow that is IOP-independent. If a cleft or separation is created in the ciliary muscle, an IOP-dependent pathway can be created secondary to ciliary body detachment [52]. This section will highlight devices that augment suprachoroidal flow.

The CyPass Microstent (Alcon Laboratories, Fort Worth, TX, USA) was a stent that was implanted through a clear corneal incision as an ab interno procedure during cataract surgery into the suprachoroidal space [53]. This device was a trabecular meshwork bypass device that was made up of a rigid, nondegradable, biocompatible polyimide. This implant had three retention rings intended to prevent slippage or drifting [52]. In 2018, CyPass was withdrawn from the market followed by FDA recall of the stent due to COMPASS-XT results showing a higher loss of endothelial cells in those undergoing cataract surgery with CyPass compared to cataract surgery alone [54].

Table 5 summarizes the characteristics and methodology of CyPass randomized controlled trials.

Ahmed et al. [26] compared the corneal endothelial density and safety after a MIGS procedure with three different devices. This randomized controlled trial compared iStent inject, Hydrus Microstent, and CyPass Microstent in mild-to-moderate primary open-angle glaucoma patients. Patients received one of these MIGS implants with phacoemulsification or standalone phacoemulsification (control). The 5-year end points compared the implant and control groups with endothelial cell loss (ECL) and mean endothelial cell density (ECD). Eyes in the iStent inject + phacoemulsification and control groups had significant ECL at 60 months (9.4% vs. 6.3%, respectively). Eyes in the CyPass + phacoemulsification and control group also had significant ECL at 60 months (27.2% vs. 10.0%, respectively). During the 5-year postoperative period, the iStent inject showed similar ECD and ECL compared to control eyes that underwent only phacoemulsification, whereas the Hydrus group and the CyPass group showed greater damage as assessed by ECL and ECD compared to control eyes with phacoemulsification only [26].

### 3.5. Other Devices That Show Potential Promise but Do Not Have Published RCTs

The iStent Supra (Glaukos, Laguna Hills, CA, USA) is a stent designed to enable drainage through the suprachoroidal space and to provide more stable retention that decreases fibrosis due to its biocompatible heparin-coated polyethersulfone ridged tube. It uses a curved design to follow the anatomical contour of the suprachoroidal space and is designed as an ab interno implant through a clear corneal incision [56]. This device is most appropriate for those with mild-to-moderate POAG as a standalone procedure or combined with phacoemulsification. This device has a favorable safety profile; no reports of suprachoroidal hemorrhage, hypotony, or hyphema have been reported [57].

The STARflo (iSTAR Medical, Wavre, Belgium) is a flexible, single continuous sheet with an anvil-like head that helps prevent extrusion. The STAR material is made up of microporous silicone and designed with hollow spheres to augment the eye’s natural uveoscleral outflow. The surgery involves an ab externo approach to increase suprachoroidal outflow. A scleral flap is made with the device head positioned in the anterior chamber with the body of the device positioned between the sclera and the choroid [55]. Transient, mild IOP peaks may occur between the fourth and twelfth postoperative weeks. Other postoperative complications may include hyphema and fibrin formation in the anterior chamber, which resolve after the first month [58].

The Biostent (IANTREK, White Plains, NY, USA) is a highly permeable, acellular scleral allograft to structurally reinforce the cyclodialysis cleft opening. Scleral allografts offer long-term durability and, due to their biomechanical properties, resemble the native sclera, lowering the risk of fibrosis and foreign body reaction [59]. The Biostent is inserted in the eye using a cyclodialysis cannula for an ab interno supraciliary approach. A cyclodialysis cannula detaches the ciliary body from the scleral spur. This surgical technique along with a scleral allograft bio-scaffold enhances uveoscleral outflow, reducing the IOP [59]. No adverse effects such as stent migration, hyphema, or corneal edema were noted at twelve months with this device [60].

AlloFlo (IANTREK, White Plains, NY, USA) is a flexible, 5 mm conforming allogenic implant made from scleral allograft homologous acellular matrix using high-precision microtrephination. This design enables a bio-scaffolded supraciliary reservoir with durable structural supplementation, eliminating the need for exogenous hardware and providing a basis for aqueous conductivity and outflow [39]. An ab interno cyclodialysis is created by the surgeon to enhance aqueous outflow, followed by positioning of AlloFlo inside the cleft. There have been minimal postoperative complications from this device such as iritis, intraocular inflammation, and implant migration due to the bio tissue ensuring no mismatch between the implant and the surrounding tissue [61].

MINIject (iSTAR Medical, Wavre, Belgium) is a suprachoroidal implant made up of the same material as STARflo, providing minimal tissue reaction. It has 27 μm spherical pores allowing for enhanced aqueous outflow and two-thirds of the implant is empty space. The device is inserted via an ab interno approach through a 2 mm clear corneal incision and inserted in the nasal quadrant. The device is flexible, and thus it conforms to the shape of the eye and enabling suprachoroidal flow. A green ring is positioned 0.5 mm from the tip of the device allowing for accurate positioning in the anterior chamber. The device is released by retracting the supportive sheath [62]. MINIject has demonstrated a favorable safety profile with minimal complications such as postoperative hyphema and cystoid macular edema that was self-limiting [63].

### 3.6. Treatment of the Ciliary Body

Aqueous humor is synthesized by the ciliary processes, and various procedures that target the ciliary body can benefit IOP lowering through multiple mechanisms. These mechanisms include a decrease in the production of aqueous humor and an increase in uveoscleral outflow due to the prevention of the contraction of the ciliary muscle [64].

Micropulse transscleral laser (IRIDEX Corporation, Mountain View, CA, USA) treatment aims to reduce the IOP by targeting the ciliary body with minimal coagulative necrosis. Short repetitive laser pulses are applied, followed by periods of rest, to the ciliary epithelium that is pigmented. The minimal coagulative necrosis induced by this process leads to decreased aqueous humor production and thus lowers the IOP [65]. The laser also causes contraction of the scleral spur, altering the configuration of the trabecular meshwork, increasing outflow via this pathway [66]. Micropulse may be recommended to those with POAG or refractory glaucoma. The probe is placed on the limbus of the eye, and a 180-degree sweeping motion is made for treatment. Adverse effects may include cystoid macular edema, transient anterior chamber inflammation, transient hypotony, hyphema, and, rarely, phthisis bulbi. Balendiran et al. [67] compared the long-term effectiveness and safety outcomes of two treatment dosages of the micropulse transscleral laser therapy in a single-blinded randomized controlled trial. A total of 19 patients with POAG were randomized into two groups: 8 to a 100 s group and 11 to a 120 s group. The baseline IOP of the 100 s group was 20.0 ± 2.45 mmHg compared to 21.09 ± 3.94 mmHg in the 120 s group. After 12 months, the mean IOP reduction in the 100 s group was 7.3 ± 4.2 mmHg compared to 9.0 ± 4.8 mmHg in the 120 s group. The authors concluded that the 120 s group achieved greater IOP control compared to the 100 s group [67].

Endoscopic cyclophotocoagulation (Endo Optiks, Little Silver, NJ, USA) is an ab interno procedure that involves direct visualization and treatment of ciliary processes. The procedure involves a clear corneal incision, and a probe is placed beneath the iris to directly visualize the ciliary process while the laser is applied. This procedure limits injury to the ciliary processes and their capillary bed whilst causing sparing damage, if any, to the ciliary muscle and stroma. With a curved probe, a single incision allows for approximately 270 degrees of treatment. This incision may be placed either temporally or superiorly near the limbus and is about 2.0 mm in width. If more treatment is desired, a second incision may be placed 180 degrees away from the initial wound for a complete 360-degree treatment and additional IOP lowering [68]. A “popping” sound indicates over-treatment of the ciliary processes and should be avoided. Endoscopic photocoagulation can be performed as a standalone procedure or in combination with phacoemulsification. Postoperative complications of endoscopic photocoagulation may include pain, diplopia, hypotony, phthisis bulbi, and cystoid macular edema [69]. Lima et al. [31] found, in a randomized controlled trial comparing the safety and effectiveness of phacoemulsification and endoscopic photocoagulation with phacoemulsification and endoscopic photocoagulation and iStent inject in open-angle glaucoma patients, that both groups achieved substantial IOP reduction at 1 year. However, the group with iStent inject achieved a greater reduction in IOP and a lower proportion of those on glaucoma medications [31].

Ultrasound cycloplasty (EyeTechCare, Rillieux-la-Pape, France) decreases the aqueous production by treating the distal portion of the ciliary body. This is accomplished by application of a high-intensity focused ultrasound that induces thermal necrosis confined to the distal portion of the ciliary body that contains the ciliary processes, the functional unit for aqueous humor production. This technique also increases uveoscleral aqueous humor outflow by increasing the intra-scleral outflow spaces [70]. Ultrasound cycloplasty may be used in those with advanced or refractory glaucoma and may be used as a standalone procedure or with phacoemulsification. A coupling cone is placed centrally on the patient’s eye followed by the treatment probe being inserted into the cone. The transducers are activated by maintaining constant pressure on the foot pedal. Adverse effects may include anterior chamber inflammation, early and recurrent cataract development/progression, and hypotony with choroidal detachment [71]. Torky et al. [72] compared ultrasound cycloplasty and cataract extraction (UCP + CE) to cataract extraction (CE) in a randomized controlled trial. A total of 61 patients with open-angle glaucoma were randomized, 31 to UCP + CE and 30 to CE. The baseline IOP in the UCP + CE and CE group was 24 mmHg. The mean IOP-lowering effect in the UCP + CE group was 7 mmHg compared to 2 mmHg in the CE group. Efficacy in this study was defined as >20% IOP reduction; 68% of the UCP + CE group was able to achieve this compared to 17% of the CE group, demonstrating the efficacy of UCP + CE [72].

### 3.7. Subconjunctival Space

Conjunctival bleb-forming procedures (minimally invasive bleb surgery): This procedure aims to reduce IOP by creating a new subconjunctival pathway. These devices create a small channel for aqueous humor drainage via the subconjunctival space by forming a blister-like fluid collection (bleb) on the surface of the eye, allowing for drainage [73].

EX-PRESS Glaucoma Filtration Device (Alcon Laboratories, Fort Worth, TX, USA) is a subconjunctival filtering device that forms a bleb-like trabeculectomy. The amount of fluid exit or filtering is managed by varying the lumen diameter, which is either 50 μm or 200 μm. The smaller lumen delivers a more regulated, slower outflow compared to the larger lumen unit, potentially decreasing the risk of post-surgical hypotony. A limbal-based conjunctival incision is made to insert the EX-PRESS device under a partial-thickness scleral flap, into the anterior chamber, similarly to a trabeculectomy, to divert aqueous humor from the anterior chamber into the subconjunctival space. The scleral flap is closed with sutures that can be titrated postoperatively to modulate aqueous outflow and maintain an optimal IOP [74,75]. This device is most appropriate for patients with uncontrolled glaucoma, including those who have failed prior medical and surgical treatments. Arimura et al. [76] found in a randomized controlled trial comparing the postoperative complications of EX-PRESS to trabeculectomy that EX-PRESS had lower postoperative complications such as anterior chamber inflammation, hypotony, and choroidal detachment. Similarly, Beltran-Agullo et al. [77] compared visual recovery following EX-PRESS to that of trabeculectomy and found those who underwent EX-PRESS recovered vision faster and were less likely to lose ≥2 Snellen lines due to complications such as cataract growth.

Table 6 summarizes the characteristics and methodology of EX-PRESS randomized controlled trials.

The XEN 45 Gel Stent (Allergan–AbbVie, Irvine, CA, USA) is supplied preloaded in an injector with a 27-gauge, double-beveled needle for ab interno placement. This creates a controlled channel for aqueous humor to drain from the anterior chamber into the subconjunctival space [83]. The implant is a 6 mm hydrophilic, crosslinked porcine gelatin stent engineered to provide a fixed outflow resistance of approximately 6–8 mmHg under normal aqueous production rates, eliminating the need for a valve mechanism [84]. By forming a new subconjunctival filtration pathway that bypasses the trabecular meshwork, the stent facilitates steady aqueous egress and absorption by surrounding tissues [82]. Although not FDA-labeled for this purpose, surgeons commonly pair the procedure with mitomycin C to reduce postoperative scarring. The XEN 45 is indicated for POAG and refractory glaucoma, particularly in patients who have failed prior surgery or whose condition remains uncontrolled on maximally tolerated medical therapy.

The bulbar conjunctiva is lifted in the superotemporal quadrant, and a preloaded injector is inserted through the conjunctiva and advanced between the tenon capsule and the conjunctiva. The needle is guided into the anterior chamber, parallel to the iris, before releasing the XEN 45 Gel Stent. A subtenon injection of mitomycin C is administered at the end of the exam to minimize bleb fibrosis. The XEN 45 Gel Stent can be placed in phakic or pseudophakic eyes as a standalone procedure. Postoperative complications may include transient IOP spikes, transient hyphema, choroidal effusion, hypotony, and bleb fibrosis [85]. Sheybani et al. compared the efficacy of the XEN stent to trabeculectomy in a randomized controlled trial. A total of 158 patients with open-angle glaucoma were randomized: 107 to the XEN 45 Gel Stent and 51 to trabeculectomy. The baseline IOP was 23.1 ± 5.8 mmHg in the XEN 45 Gel Stent group compared to 22.6 ± 5.7 mmHg in the trabeculectomy group. After 12 months, the mean IOP in the XEN 45 Gel Stent group was 14.4 ± 4.1 mmHg compared to 11.8 ± 3.5 mmHg in the trabeculectomy group. Efficacy in this study was defined as ≥20% IOP reduction; 62.1% of the XEN group achieved this compared to 68.20% of the trabeculectomy group. The authors concluded there was no statistically significant difference between these two treatments [86].

The PRESERFLO MicroShunt (Santen Pharmaceutical Co., Osaka, Japan) is a small, flexible tube made of a flexible polymer aimed at minimizing inflammation, scarring, and fibrosis [87]. The PRESERFLO MicroShunt is an implant that requires an ab externo approach that involves a subconjunctival incision and a scleral flap that creates a pocket for the device, which is followed by the implantation of the device’s distal end, which is positioned under the conjunctiva and the Tenon’s capsule. This diverts the fluid from the anterior chamber into the subconjunctival space, creating a bleb and thereby lowering IOP [87,88]. This procedure can be performed as a standalone surgery or combined with cataract extraction with phacoemulsification. The 1 mm “wing” of the shunt is positioned 4.5 mm from the flat end and buried in the scleral pocket to prevent the shunt’s dislocation into the anterior chamber. This procedure is usually recommended in individuals with moderate-to-severe POAG. Postoperative complications may include transient hypotony, choroidal effusion, hyphema, keratitis, and bleb fibrosis [89]. Scheres et al. compared the effectiveness and cost-effectiveness of MicroShunt implantation to trabeculectomy and concluded that it was unclear whether the MicroShunt was more cost effective [90]. Atik et al. [91] also performed a randomized controlled trial comparing the cost-effectiveness of MicroShunt implantation to trabeculectomy and found trabeculectomy to be superior to the MicroShunt in terms of surgical management of POAG. Panarelli et al. [92] compared the efficacy of the MicroShunt to trabeculectomy in a prospective, randomized, multi-center trial. A total of 527 patients with primary open-angle glaucoma were randomized, 395 to the MicroShunt and 132 to trabeculectomy. The mean IOP in the MicroShunt group was 21.1 ± 4.9 mmHg compared to 21.1 ± 5.0 mmHg in the trabeculectomy group. After 24 months, the mean IOP in the MicroShunt group was 13.9 ± 3.9 mmHg compared to 10.7 ± 3.7 mmHg in the trabeculectomy group. In this study, efficacy was defined as ≥20% IOP reduction; 50.6% of the MicroShunt group were able to achieve this compared to 64.4% of the trabeculectomy group, demonstrating a higher surgical success rate in the trabeculectomy group [92].

## 4. Discussion

MIGS has redefined the surgical management of glaucoma by offering reduced complication rates, a shorter recovery time, and earlier intervention, making it an increasingly popular choice, particularly in individuals who are generalists or non-glaucoma specialty-trained ophthalmologists [93]. However, MIGS is not without limitations and challenges. One of the limitations MIGS faces is efficacy; it is most effective in those with mild-to-moderate glaucoma. The capability for significant IOP reduction in severe glaucoma is not as substantial compared to traditional filtering surgeries such as trabeculectomies or glaucoma drainage devices [94]. Hence, patients that require large IOP reductions such as 40% from baseline, which are commonly needed in advanced glaucoma, are not suitable candidates for MIGS procedures. The second important challenge that all MIGS devices face is the lack of long-term efficacy data. Traditional filtering surgeries have extensive long-term data, whereas most MIGS studies have shorter follow-up periods [10]. Currently we do not have information of long-term efficacy of MIGS and the need for follow-up and additional procedures to maintain IOP long term [95]. It is also prudent for eye care providers to understand the financial burden MIGS may have on patients. The cost of MIGS devices and surgical fees may be initially expensive, especially when compared to traditional glaucoma surgeries and selective laser trabeculoplasty [96]. However, the long-term cost–benefit of MIGS is arguably better than that of the long-term costs of topical therapy with IOP-lowering medications or management of potential complications of more invasive surgeries. Although there is some initial cost–benefit research that has published these findings [24,96], more extensive cost–benefit analysis is needed, including all glaucoma treatment modalities and their benefits to quality of life and visual outcomes. It will also be interesting to investigate if MIGSs are indeed utilized by general ophthalmologists more than glaucoma specialists.

Some of these limitations will help shape the future of MIGS in terms of research, innovation, and glaucoma management [97]. New devices and stents are on the horizon, targeting different aqueous humor outflow pathways and improving on the IOP-lowering capabilities of MIGS. There is ongoing research into MIGS devices that have the capability of delivering medication and performing surgical interventions directly into the target tissue to enhance IOP control further [98]. The incorporation of imaging technologies, such as optical coherence tomography angiography, enables enhanced precision and safety in MIGS procedures, facilitating more accurate device placement and improved assessment of surgical outcomes [97,98]. This leads to increased efficacy and fewer postoperative complications. The use of artificial intelligence is rising amongst various healthcare platforms. Artificial intelligence can assist surgeons in patient selection, device/technique optimization, pre- and postoperative risks, and help predict the success rate of the procedure. Artificial intelligence predicts this by including factors such as patient history, visual field loss, and optic nerve damage severity, amongst other factors [99].

One of the shortcomings was our inability to objectively compare postoperative transient events. MIGS devices are fundamentally divided by the outflow facility they influence and thus may vary in their ability to lower IOP. The differences in IOP-lowering effects, implant location, number of implants, implant size, bleb-forming or non-bleb-forming, and drainage modalities are among the features that determine the suitability of these devices at different stages of glaucoma. Thus, the type of MIGS device influences the transient postoperative events, and describing them descriptively was the best that could be performed.

Current research is focused on long-term evaluation and comparison of MIGS devices to conventional surgeries. The outcomes of these studies will shed light on efficacy, safety, and long-term IOP control with the MIGS devices [100,101,102]. The studies will also uncover the various strengths and limitations of the new technologies and procedures. Last but not least, there is tremendous interest in learning about perceived and actual quality of life improvements to guide our management strategies and ensure a patient-centered approach to glaucoma care [93,100].

## 5. Conclusions

Glaucoma is one of the leading causes of irreversible blindness globally, and the only proven method for halting disease progression is IOP lowering. For glaucoma patients where pharmacological medication, laser treatment, and surgery are not safe and effective, MIGS may offer a tailored approach to help reduce the IOP. MIGS plays a pivotal role in glaucoma management, by providing sustained IOP reduction, fewer postoperative complications, overall cost-effectiveness, and ultimately better patient quality of life. This enormous potential of MIGS devices will need to be substantiated further with more long-term data on the safety of implants and efficacy in lowering and maintenance of intraocular pressure.

## Figures and Tables

**Table 3:** Characteristics and methodology of Hydrus Microstent randomized controlled trials.

Study	Number of	Age of	Length of	Baseline IOP	IOP-Lowering Effect	Secondary	Efficacy/
(Name)	Participants	Participants (Years)	Study (Months)	(mmHg)	(mmHg)/Outcome	Outcome	Rate of Success
Ahmed, 2020 [25]	75	66.9 ± 10.0	12	19.0 ± 3.9 mmHg	17.3 ± 3.3	46.6% (medication	30.1% (IOP ≤ 18 mmHg
(COMPARE)	(Hydrus)	(Hydrus)		(Hydrus)	(Hydrus)	reduction; Hydrus)	without treatment; Hydrus)
	77	66.5 ± 9.5		19.1 ± 3.6 mmHg	19.2 ± 2.4	24% (medication	9.3% (IOP ≤ 18 mmHg
	(iStent)	(iStent)		(iStent)	(iStent)	reduction; iStent)	without treatment; iStent)
Ahmed, 2021 [35]	369	not reported	36	17.9 ± 3.1	16.7 ± 3.1	0.4 ± 0.8 (number of	62%
(HORIZON)	(Hydrus)			(Hydrus)	(Hydrus)	medications used; Hydrus)	(≥20% IOP reduction; Hydrus)
	187	not reported		18.1 ± 3.1	17.0 ± 3.4	0.8 ± 1.0 (number of	41.10%
	(CE)			(CE)	(CE)	medications used; CE)	(≥20% IOP reduction; CE)
Ahmed, 2024 [26]	69 ± 7.8	178	60	not reported	not reported	2099 ± 430	not reported
	(iStent inject treatment)	(iStent inject treatment)				(ECL cells/mm^2^)	
	69.3 ± 7.0	49		not reported	not reported	2103 ± 419	not reported
	(iStent inject control)	(iStent inject control)				(ECL cells/mm^2^)	
	71.1 ± 7.9	369		24.4 ± 2.8	not reported	1967 ± 522	not reported
	(Hydrus treatment)	(Hydrus treatment)		(Hydrus treatment)		(ECL cells/mm^2^)	
	71.2 ± 7.6	187		24.5 ± 3.0	not reported	2117 ± 442	not reported
	(Hydrus control)	(Hydrus control)		(Hydrus control)		(ECL cells/mm^2^)	
	69.4 ± 7.9	215		24.5 ± 2.9	not reported	1931 ± 517	not reported
	(CyPass treatment)	(CyPass treatment)		(CyPass treatment)		(ECL cells/mm^2^)	
	70.8 ± 7.5	67		24.8 ± 3.1	not reported	2189 ± 375	not reported
	(CyPass control)	(CyPass control)		(CyPass control)		(ECL cells/mm^2^)	

Legend: CE: cataract extraction, ECL: endothelial cell loss.

**Table 4:** Characteristics and methodology of ab interno canaloplasty randomized controlled trials.

Study	Number of	Age of	Length of	Baseline IOP	IOP-Lowering Effect	Secondary Outcomes	
(Name)	Participants	Participants (Years)	Study (Months)	(mmHg)	(mmHg)/Outcome	(Number of Glaucoma Medications)	Efficacy/Rate of Success
Ye, 2025 [50]	26	44.2 ± 14.5	24	30.8 ± 10.7	14.1 ± 3.3	0.2 ± 0.5	92%
	(PCP)	(PCP)		(PCP)	(PCP)	(PCP)	(≥20% IOP reduction; PCP)
	26	44.7 ± 10.0		28.6 ± 11.8	22.1 ± 13.6	0.7 ± 1.2	77%
	(ABiC)	(ABiC)		(ABiC)	(ABiC)	(ABiC)	(≥20% IOP reduction; ABiC)
Yin, 2024 [46]	39	41 ± 15	12	25.6 ± 10.1	16.0 ± 3.1	0.6 ± 1.2	75% (IOP ≤ 21 mmHg and
	(GATT)	(GATT)		(GATT)	(GATT)	(GATT)	non-use of glaucoma medications; GATT)
	38	41 ± 13		24.9 ± 10.0	19.0 ± 5.2	0.9 ± 1.3	56% (IOP ≤ 21 mmHg and
	(ABiC)	(ABiC)		(ABiC)	(ABiC)	(ABiC)	non-use of glaucoma medications; ABiC)

Legend: GATT: gonioscopy-assisted transluminal trabeculectomy, ABiC: ab interno canaloplasty, PCP: penetrating canaloplasty.

**Table 5:** Characteristics and methodology of CyPass randomized controlled trials.

Study	Number of	Age of	Length of	Baseline IOP	IOP-Lowering Effect		
(Name)	Participants	Participants (Years)	Study (Months)	(mmHg)	(mmHg)/Outcome	Secondary Outcomes	Efficacy/Rate of Success
Ahmed, 2024 [26]	69 ± 7.8	178	60	not reported	not reported	2099 ± 430	not reported
	(iStent inject treatment)	(iStent inject treatment)				(ECL cells/mm^2^)	
	69.3 ± 7.0	49		not reported	not reported	2103 ± 419	not reported
	(iStent inject control)	(iStent inject control)				(ECL cells/mm^2^)	
	71.1 ± 7.9	369		24.4 ± 2.8	not reported	1967 ± 522	not reported
	(Hydrus treatment)	(Hydrus treatment)		(Hydrus treatment)		(ECL cells/mm^2^)	
	71.2 ± 7.6	187		24.5 ± 3.0	not reported	2117 ± 442	not reported
	(Hydrus control)	(Hydrus control)		(Hydrus control)		(ECL cells/mm^2^)	
	69.4 ± 7.9	215		24.5 ± 2.9	not reported	1931 ± 517	not reported
	(CyPass treatment)	(CyPass treatment)		(CyPass treatment)		(ECL cells/mm^2^)	
	70.8 ± 7.5	67		24.8 ± 3.1	not reported	2189 ± 375	not reported
	(CyPass control)	(CyPass control)		(CyPass control)		(ECL cells/mm^2^)	
Reiss, 2019 [54]	215	69.4 ± 7.9	60	24.4 ± 2.8	8.4	1/9	not reported
(COMPASS-XT)	(CE + CyPass)	(CE + CyPass)		(CE + CyPass)	(7.8–8.9; CE + CyPass)	(adverse effect)	
	67	70.8 ± 7.5		24.5 ± 3.0	8	4/67	not reported
	(CE)	(CE)		(CE)	(6.8–9.2; CE)	(adverse effect)	
Vold, 2016 [55]	374	>45	24	24.4 ± 2.8	↓7.4	none reported	77%
	(CyPass)	(CyPass and control)		(CyPass)	(CyPass)		(≥20% IOP reduction)
	131	>45		24.5 ± 3.0	↓5.4	none reported	60%
	(control)	(CyPass and control)		(control)	(CE)		(≥20% IOP reduction)

Legend: CE: cataract extraction, ECL: endothelial cell loss.

**Table 6:** Characteristics and methodology of EX-PRESS randomized controlled trials.

Study	Number of	Age of	Length of	Baseline IOP	IOP-Lowering Effect		
(Name)	Participants	Participants (Years)	Study (Months)	(mmHg)	(mmHg)/Outcome	Secondary Outcomes	Efficacy/Rate of Success
Dahan, 2012 [78]	15	65.4 ± 13.7	23.6	31.1 ± 14.2	16.2 ± 1.5	33% (post-op complications;	47.5% (IOP ≤ 18 mmHg;
	(trabeculectomy)	(trabeculectomy and EX-PRESS)		(trabeculectomy)	(trabeculectomy)	trabeculectomy)	trabeculectomy)
	15	65.4 ± 13.7		28.1 ± 9.0	15.7 ± 1.8	27% (post-op complications;	81.8% (IOP ≤ 18 mmHg;
	(EX-PRESS)	(trabeculectomy and EX-PRESS)		(EX-PRESS)	(EX-PRESS)	EX-PRESS)	EX-PRESS)
de Jong, 2009 [79]	40	62.3 ± 14.5	12	22.8 ± 8.0	12.0 ± 2.7	2.8 ± 1.3 (medication reduction;	76.9% (IOP ≤ 18 mmHg;
	(Ex-PRESS)	(EX-PRESS)		(EX-PRESS)	(EX-PRESS)	EX-PRESS)	EX-PRESS)
	40	68.9 ± 11.5		21.5 ± 5.6	13.9 ± 4.3	3.0 ± 0.9 (medication reduction;	50% (IOP ≤ 18 mmHg;
	(trabeculectomy)	(trabeculectomy)		(trabeculectomy)	(trabeculectomy)	trabeculectomy)	trabeculectomy)
de Jong, 2011 [80]	39	68.6 ± 11.5 (trabeculectomy)	60	21.3 ± 5.6	11.3 ± 1.9	none reported	none reported
	(trabeculectomy)			(trabeculectomy)	(trabeculectomy)		
	39	62.4 ± 14.7 (EX-PRESS)		22.8 ± 8.0	11.5 ± 2.9	none reported	none reported
	(EX-PRESS)			(EX-PRESS)	(EX-PRESS)		
Netland, 2014 [81]	59	69.4 ± 11.6	24	25.1 ± 6.0	14.7 ± 4.6	none reported	83% (IOP ≤ 18 mmHg;
(XVT Study)	(EX-PRESS)	(EX-PRESS)		(EX-PRESS)	(EX-PRESS)		EX-PRESS)
	61	67.8 ± 10.4		26.4 ± 6.9	14.6 ± 7.1	none reported	79% (IOP ≤ 18 mmHg;
	(trabeculectomy)	(trabeculectomy)		(trabeculectomy)	(trabeculectomy)		trabeculectomy)
Wagdy, 2021 [82]	14	42–55	12	29–34	14–16.5	none reported	IOP ≤ 21 mmHg without
	(EX-PRESS)	(EX-PRESS and SST + MMC)		(EX-PRESS)	(EX-PRESS)		treatment; 62.3% (EX-PRESS)
	14	42–55		29.75–33.25	14.75–23.5	none reported	IOP ≤ 21 mmHg without
	(SST + MMC)	(EX-PRESS and SST + MMC)		(SST + MMC)	(SST + MMC)		treatment; 66.6% (SST + MMC)

Legend: SST—subscleral trabeculectomy, MMC—mitomycin C.

**Figure A1:**
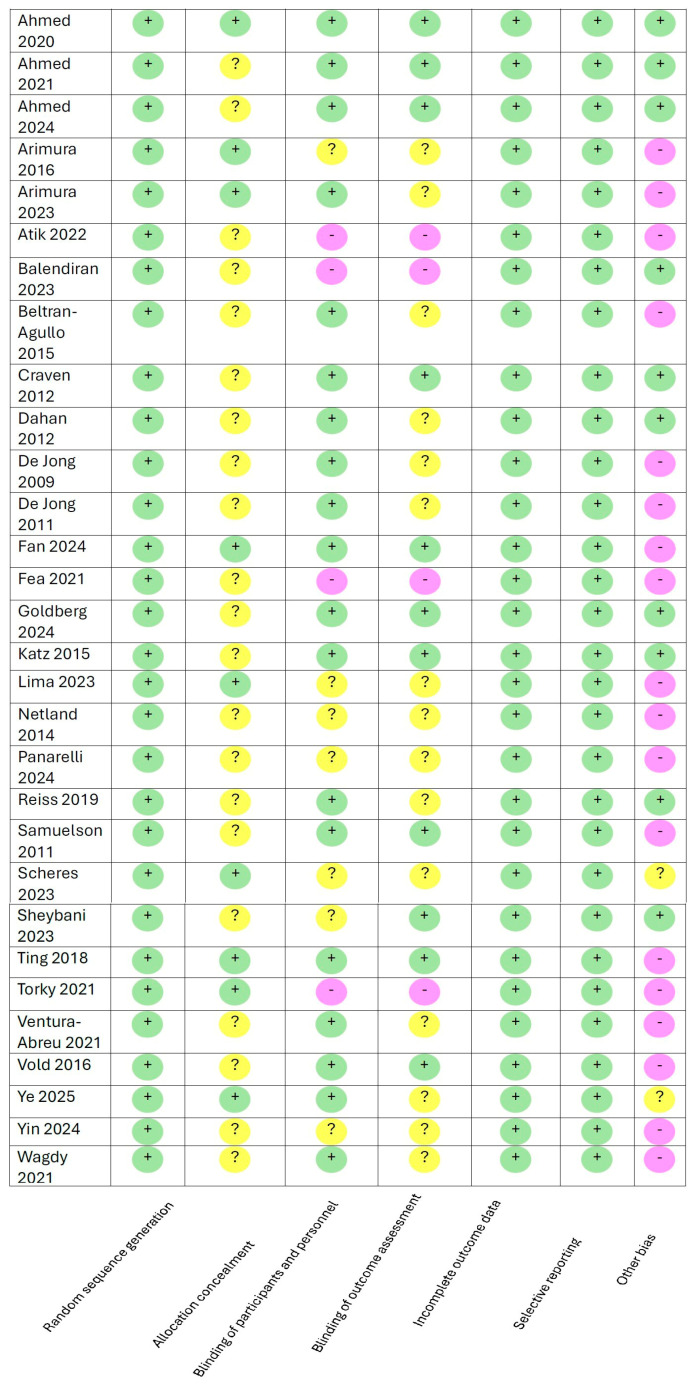

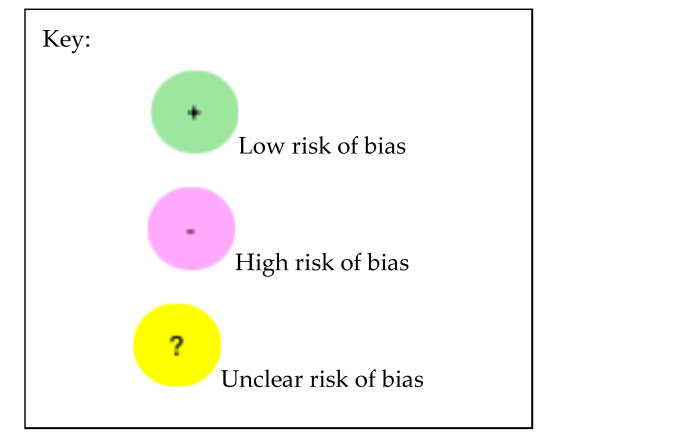
Risk of bias assessment of the studies on the basis of Cochrane Collaboration’s tool for assessing risk of bias [24,25,26,27,28,29,30,31,32,35,40,41,43,46,50,54,55,67,72,76,77,78,79,80,81,82,86,90,91,92].

## Data Availability

No new data were created or analyzed in this study. Data sharing is not applicable to this article.

## Abbreviations

The following abbreviations are used in this manuscript:

ABiC	Ab Interno Canaloplasty
CE	Cataract Extraction
CP	Canaloplasty
IOP	Intraocular Pressure
ECD	Endothelial Cell Density
ECL	Endothelial Cell Loss
FDA	Food and Drug Administration
GATT	Gonioscopy-Assisted Transluminal Trabeculectomy
MIGS	Minimally Invasive Glaucoma Surgery
MMC	Mitomycin C
PCP	Penetrating Canaloplasty
Phaco-AIT	Trabectome with Cataract Surgery
Phaco-trabeculectomy	Trabeculectomy with Mitomycin C And Cataract Surgery
POAG	Primary Open-Angle Glaucoma
RCT	Randomized Controlled Trial
SST	Subscleral Trabeculectomy
UCP + CE	Ultrasound Canaloplasty and Cataract Extraction
VFQOfL	Visual Function Quality Of Life
